# Free Fatty Acid and α-Lactalbumin-Oleic Acid Complexes in Preterm Human Milk Are Cytotoxic to Fetal Intestinal Cells *in vitro*

**DOI:** 10.3389/fnut.2022.918872

**Published:** 2022-07-05

**Authors:** Katherine E. Chetta, Danforth A. Newton, Carol L. Wagner, John E. Baatz

**Affiliations:** Division of Neonatology, Department of Pediatrics, Darby Children's Research Institute, Medical University of South Carolina, Charleston, SC, United States

**Keywords:** human milk, storage, free fatty acid, HAMLET, cytotoxicity, oleic acid, arachidonic acid, necrotizing enterocolitis

## Abstract

Human milk, the best enteral selection for a preterm infant, becomes altered during freezing and soluble free fatty acid is generated over time. Free fatty acids may form complexes, such as the oleic acid-bound protein called HAMLET (human α-lactalbumin made lethal to tumor cells). We determined the *in vitro* biological activity of preterm human milk protein-oleic complexes (HAMLET-like complexes) and tested the hypothesis that laboratory-synthesized HAMLET exhibits cytotoxicity in human immature epithelial intestinal cell culture. Thirty-four milk samples from 15 mothers of hospitalized preterm infants were donated over time. Milk fractions were tested repeatedly for FHs 74 Int and HIEC-6 fetal cell cytotoxicity, using a sensitive viability assay. Protein and fatty acid identities were confirmed by Western blot, high performance liquid chromatography, and mass spectrometry. Cytotoxicity of intestinal cells exposed to milk increased respective to milk storage time (*p* < 0.001) and was associated with free oleic acid (*p* = 0.009). Synthesized HAMLET was cytotoxic in cultures of both lines. Preterm milk samples killed most cells in culture after an average 54 days in frozen storage (95% C.I. 34–72 days). After prolonged storage time, preterm milk and HAMLET showed a degree of cytotoxicity to immature intestinal cells in culture.

## Introduction

Health outcomes for infants receiving their own mother's milk are associated with superior health benefits when compared to infants receiving donor milk or formula (1–4). The primary concerning outcomes from donor milk are slower somatic growth and less optimal neurodevelopment (1, 3, 5–7). In contrast to fresh mother's milk, donor milk is typically stored for months before use by preterm infants (8). It is known that milk fats release free fatty acids (FFA) over time (9), even while frozen at −20°C (10, 11), but the biologic relevance of milk storage time has received relatively less attention than the effects of pasteurization (8). It is well-known that FFA can be cytotoxic to various cell types (12–17), but FFA that accumulate in stored human milk may have more biological relevance than previously considered.

Severe gastrointestinal morbidities are common in preterm infants, including necrotizing enterocolitis (NEC) with an incidence of 5–7% for infants < 1,500 g birth weight (18), and spontaneous intestinal perforations (SIP) affect up to 20% of infants born <1,000 g (19). NEC and SIP are the most common reasons for gastrointestinal surgeries in the neonatal intensive care units and can have mortality rates exceeding 50% (18). Risk factors underlying these disease processes are still unclear, but previous work has suggested FFA may play a role. A study by Penn et al. reported that in digested milk, FFA could cause cytotoxicity in bovine epithelial cells (20, 21). Free oleic acid, specifically, has been shown to cause cytotoxicity in rodent intestinal epithelial cells, and various other cell types (12, 13, 16, 22). It has also been demonstrated that emulsified oleic acid induces intestinal colitis in rodent models (12, 14, 21).

Fresh milk does not have significant levels of FFA, due to two main features: (1) fatty acids are present as triglycerides, in which the fatty acid is not free but instead is covalently linked to the glycerol skeleton, and (2) the membrane of the milk fat globule, a unique triple phospholipid membrane is serving as a protective cover of encapsulated fat, thus preventing lipase-catalyzed hydrolysis of triglycerides (23). Lipases in human milk are important during the digestive phase, to make milk fat readily available for absorption by mature enterocytes in a controlled fashion using bile salts and micelles at the enterocyte interface (23). However, the exact mechanism of transport of FFA into the enterocyte is not well-known in the neonate, and both protein-independent (diffusion) and protein-dependent methods have been proposed (24, 25). The mechanisms of endocytosis for FFA-bound proteins are relatively unknown in the immature epithelium of the preterm intestine.

This study includes human milk from mothers of preterm infants and quantifies the levels of 15 FFA species in their stored samples. Milk was tested for cytotoxicity in 2 fetal intestinal epithelial cell cultures and correlated to milk FFA. Furthermore, we synthesized a milk protein-fatty acid complex called human α-lactalbumin made lethal to tumors (HAMLET), which is a lipid-protein complex of α-lactalbumin bound to oleic acid and tested it for cytotoxicity in fetal intestinal cell culture (26, 27). HAMLET has been shown to cause apoptosis in undifferentiated cell types (28). In storage conditions, FFA in human milk may spontaneously generate HAMLET or HAMLET-like complexes. We hypothesized that (1) fetal intestinal cell death observed after preterm milk exposure is related to the number of days the milk was in frozen storage, (2) the mechanism of cell death is due primarily to increased formation of *HAMLET-like* complexes (oleic acid bound non-specifically to milk protein), and (3). HAMLET would be cytotoxic to human fetal intestinal cells (FHs 74 Int and HIEC-6). This study explores FFA-related cytotoxicity specific to immature human intestinal epithelium.

## Methods

### Consent

This study was approved by the institutional review board of the Medical University of South Carolina (MUSC) #Pro00103782 on January 26, 2021. Eligible subjects were screened and consented to donate their milk.

### Subjects

During the period May 2021 to February 2022, 15 mothers of infants born <37 weeks gestational age who were providing milk to their infants were enrolled. One mother of a 39-week infant was enrolled for reference. Mothers were ineligible if they had positive screens for HIV, RPR, CMV or coronavirus 19 during prenatal screening. Mothers were instructed on how to pump, clean pumping equipment and express their milk by Lactation Specialists as standard neonatal intensive care practice. Two or three 80–100 mL samples of breastmilk were obtained from each mother depending on patient preference, spaced by at least 2-week intervals. Milk was obtained between 5 and 105 days postpartum. Before donation, samples were kept frozen at −20°C before in the hospital Nutrition Management Center, which is equipped with industrial freezers and remote temperature monitoring. The milk was thawed immediately before analysis. Banked human donor milk was obtained from the hospital's nutrition management center.

### Milk Processing

Mother's milk was thawed in 1 mL aliquots, centrifuged at 8,000 × g for 20 min (Eppendorf 5415 D, Enfield, CT), chilled for 20 min, and the fat layer removed. The skimmed portion was diluted to 10% of initial concentrations for cell incubation using calcium- and magnesium-free Hank's buffered salt solution, (HBSS, Gibco, Grand Island, NY). Ten percent concentrations were chosen based on preliminary studies, and found to be a concentration that imparted biological activity but did not interfere with cell adhesion. To obtain casein and whey fractions, skimmed milk was first acidified to 4.3 pH using 1.0N HCl and then centrifuged at 10,000 × g for 5 min, based on a previous protocol with modifications (29). The whey portion was decanted, then was pH-corrected to 6.8 with 0.25N NaOH. The casein portion was diluted by adding HBSS to obtain the starting volume of skimmed milk.

### Cell Lines

The non-transformed human cell line, FHs 74 Int (FHs 74), is an intestinal epithelial cell line originating from a female fetus of 10–13 weeks (30, 31). FHs 74 cells were cultured in Hybricare Media (ATCC, Manassas, VA) with 10% fetal bovine serum (FBS) and 10 ng/mL EGF and passaged 3–7 times under 5% CO2. To passage, cells were grown in 75 mm^2^ flasks to 70% confluence, gently removed from the plate using trypsin with 0.25% trypsin-EDTA solution, and split 1:2 or 1:3 into fresh complete media. A second fetal epithelial intestinal line, HIEC-6 (32), from a 13–17-week fetus was grown with 5% FBS in Opti-MEM reduced serum medium with GlutaMAX (Gibco, Waltham, MA) and 30 ng/mL EGF. Passages 3-7 were used for *in vitro* viability studies. A lung carcinoma cell line, A549 (33, 34), was used as a control for comparison and was grown in Dulbecco's Modified Eagle Medium (Sigma, Burlington, MA) with 10% FBS and passaged similarly.

### MTT Assay Viability

Intestinal cell lines were grown in a non-pyrogenic sterile polystyrene 96-well culture cluster plate (Costar, Corning, NY). Cells were seeded at 1 × 10^4^ cells per well and cultured in complete media overnight at 37°C and 5% CO_2_ to reach 50–70% confluence. The next day, media was removed, and the cell monolayer was gently washed twice with HBSS solution. Diluted 10% skimmed preterm milk, preterm milk casein and preterm milk whey solution in HBSS (50 μL) were separately added to wells in triplicate and incubated for 1 h. Protein-free HBSS solution was used as a negative control, and lysis solution (Promega, Madison, WI) was used as a positive (cytotoxic) control. After 1 h, 40 μL of complete media was added to each well, and incubated for 2.5 more hours, after 10 μL of 10x EZMTT™ High Sensitivity Cell Viability Assay Solution (EMD Millipore, Burlington, MA) was added to wells. This assay involves a novel mono-sulfonated tetrazolium salt (2-(2-(2-methyloxy-4-nitrophenyl)-2-(4-nitrophenyl)-2H-tetrazol-3-ium-5-y) that reacts with oxidoreductase enzymes, markers of cellular metabolic activity, and directly converts to a soluble yellow formazan, which can be measured at 450 nm by a spectrophotometer plate reader (Epoch, Gen 5 software, Biotek^®^ Instruments). This one-step process reduces intraexperimental variability. Viability >100% represents increased metabolic activity of the cells. Viability tests with HAMLET and α-lactalbumin were performed 4 independent times.

### TUNEL Assay

Fetal intestinal cells were grown on coverslips in sterile 6-well dishes. Negative control with complete media and positive controls with 1 μg/ml staurosporine to induce apoptosis were used. Cells were incubated with 1% milk concentrations for 2 h. (10% concentrations rapidly led to complete cell fragmentation; therefore 1% was used to ensure cells adhered to the slide). Media was removed and cells were gently washed with PBS. Cells were fixed with 3.7% formalin solution for 15 min at room temperature (RT), then washed x 3 with PBS solution and stored at 4°C until use. Terminal deoxynucleotidyl transferase dUTP nick end labeling (TUNEL) assay was performed on fixed cells after permeabilization with 0.1% Triton™ X-100 using the Click-iT™ TUNEL imaging assay using a Picolyl azide Alexa Fluor™ 488 probe according to manufacturer's instructions. Cells were mounted on slides using DAPI nuclear staining fixative and imaged with a fluorescent microscope.

### Free Fatty Acid Quantification

A quantitative analysis of 15 fatty acid species was performed by the MUSC Lipidomics Core Facility on samples from all 15 patients. Skimmed milk samples were tested for FFA composition at the same time as viability testing (*n* = 39). Fatty acid compositions of α-lactalbumin, HAMLET, non-diluted skim, whey (*n* = 8) and casein milk fractions (*n* = 8) were analyzed by HPLC-tandem MS/MS. The total protein content of milk was quantified before analysis by the bicinchoninic calorimetric assay (BCA Protein Assay Kit, ThermoScientific, Rockford, IL).

#### Extraction

Ammonium bicarbonate (10 mM) and 20 nmol/mL free fatty acid Internal Standard were added to each sample and mixed thoroughly *via* vortex. Extraction solution (15:85 vol/vol Isopropanol: Ethyl Acetate) was then added to each solution. The samples were vortexed and centrifuged for 5 min at 3,000 RPM (Beckman Allegra 6R Centrifuge, Indianapolis, IN). The supernatants were collected and 100 μL of formic acid was added and the extraction procedure repeated, after which, the supernatant was collected and dried under Nitrogen gas. The extract was resuspended in 150 μl Negative Ion Mobil Phase (1 mM NH_4_COOH, 0.2% vol/vol NH_4_OH in Methanol).

#### Free Fatty Acid Species Determination

Separation and identification of FFA species were performed by HPLC-MS/MS consisting of a Thermo Scientific Vanquish UHPLC system coupled to a Thermo Quantis triple quadrupole mass spectrometer equipped with an ESI probe operating in negative, selective ion monitoring mode. Chromatographic separations were obtained under a gradient elution of water and methanol using ammonium formate and ammonium hydroxide for ionization on a C18 column at ambient temperature. The mobile phase was 2 mM NH_4_OCOOH; 0.2% NH_4_OH in H_2_O 1 mM NH_4_OCOOH; 0.2% NH_4_OH in MeOH on a C18 Column (Peak Scientific, UK). FFA species analyzed with known standards were: C12:0, C14:0, C16:0, C16:1, C18:0, C18:1, C20:0, C20:1, C20:4, C22:0, C22:1, C24:0, C24:1, C26:0, C26:1.

### Alpha-Lactalbumin Purification

Alpha-lactalbumin was purified from donor human milk *via* previous methods (35). Briefly, milk was skimmed as above, and the whey fraction was collected *via* acid precipitation. Alpha-lactalbumin was separated from other whey proteins with a fast protein liquid chromatography (FPLC) system which included a glass 45/25 mm FPLC fitted chromatography column (GE Pharmacia, Uppsala, Sweden) packed with DEAE sepharose CL-6B (GE Pharmacia, Uppsala, Sweden), and coupled to a UV-1 optical unit with 280 nm absorbance (Pharmacia LKB, Sweden). For controlled mobile phase flow, a peristaltic pump set to 17.2 rpm (Minipuls2 Gilson^®^, Middleton, WI) was used to achieve a 1.5–1.8 ml/min flow rate. Protein was eluted with 10 mM Tris buffer pH 8.5 and a salt gradient of 0–1.0 M NaCl. Protein-containing fractions were then identified by SDS-PAGE gel, Western blot with Coomassie stain. Eluted fractions containing α-lactalbumin protein were combined and EDTA was added to a final concentration of 5 mM. Chelated α-lactalbumin was added to a second DEAE sepharose column CL-6B on an FLPC system and eluted with a gradient of 0–0.4 M NH_3_HCO_3_ buffer. Protein-containing fractions were detected at 280 nm and collected using a fraction collector. Protein was lyophilized for 72 h and stored at −20°C until use. Final protein solutions were analyzed with UHPLC-MS/MS to determine free fatty acid composition and found to be pure with a fatty acid content <1 nmol/100 μg protein.

### HAMLET Synthesis

Purified de-lipidated α-lactalbumin in 10 mM Tris HCl was loaded on an oleic-acid conditioned DEAE sepharose CL-6B column in the FPLC system following methods per Brinkmann et al. (35). To condition column, a slurry of 43 g of sepharose CL-6B in 100 mL of 10 mM Tris buffer, pH 8.5 was prepared. Eighty microliters of pure oleic acid (Sigma, Burlington, MA) were dissolved in 400 μL of laboratory grade 200 proof ethanol (Sigma, Burlington, MA). The oleic acid-ethanol solution was added drop wise to the sepharose solution and sonicated 5 min × 3 cycles. The oleic acid-conditioned DEAE sepharose slurry was added to a clean glass chromatography column. Unbound oleic acid was washed off using 100 mL of 1.0 M NaCl in 10 mM Tris buffer, pH 8.5. Then 10 mM Tris buffer solution (0 M NaCl) was added until the chart recorder equilibrated to the UV-1 baseline (~500 mL). Alpha-lactalbumin (30 mg) was dissolved in 5 mL of 10 mM Tris-HCl in 0.07 mM EDTA, gently mixed for 1 h then added to the column. With a NaCl gradient, and 10 mM Tris buffer 8.5, two peaks eluted at approximately 0.3 M NaCl (native form), and the bound form after 1.0 M NaCl (HAMLET). HAMLET protein was dialyzed 48 h with Spectra/Por^®^ Membrane MWCO 6-8,000, (Spectrum labs, New Brunswick, NJ) against 1L Milli-Q water, with 4 changes. Alternatively, protein was desalted with centrifugation against PBS with a 10k Ultracel membrane (Amicon^®^, Sigma, Burlington, MA) for immediate use. SDS-PAGE gel with Coomassie stain, mass spectrometry and Western blot confirmed proteins of interest. Protein was tested for cytotoxicity in FHs 74, HIEC-6, and A549 cell culture. HAMLET was quantified by BCA for mass spectrometry and lipidomic analysis.

### SDS PAGE and Western Blot

The protein peaks eluting from a DEAE cellulose column after 1.0 M NaCl (HAMLET) were analyzed by separation on 4–12% Bis-Tris NuPAGE Gel (NuPage™, Invitrogen, US) and MES running buffer, followed by Coomassie staining or Western blotting. Nitrocellulose blots were incubated with rabbit monoclonal antibody to human α-lactalbumin (Abcam, Branford, CT), and 800CW donkey anti-rabbit IR Dye labeled secondary antibody (LiCor^®^, Lincoln, NE) then read by an IR800 scanner (Odyssey-CLx-C551), CLx (LiCor^®^ Lincoln, NE) using imaging software (Image Studio v. 4.0).

### Mass Spectrometry

The MUSC Proteomics Core Facility analyzed protein in purified native α-lactalbumin and HAMLET to evaluate for homologous protein sequences by HPLC tandem mass spectrometry with Orbitrap Fusion Lumos technology coupled with a protein database search.

#### Sample Preparation

Protein was reduced in 1 mM dithiothreitol and alkylated in 5.5 mM iodoacetamide. The samples were digested with trypsin overnight at 37°C with 100 ng of trypsin. The digestion was acidified to 1% formic acid. The resulting peptides were desalted using C18 Stage Tips. Tips were conditioned with 80% acetonitrile (ACN), 5% formic acid (FA), equilibrated and loaded in 95% water, 5% FA. The peptides were desalted by washing with 95% water, 5% formic acid 5 times, and eluted with 80% ACN, 5% FA. The peptides were dried in a SpeedVac and stored at −80°C.

#### Liquid Chromatography and Mass Spectrometry Data Acquisition Parameters

Peptides were quantified using C18 staged tips with a 10 μg capacity loaded at 20%, separated and analyzed on an EASY nLC 1200 System (ThermoScientific) in-line with the Orbitrap Fusion Lumos Tribrid Mass Spectrometer (ThermoScientific) with instrument control software v. 4.2.28.14. Two μg of tryptic peptides were pressure loaded onto C18 reversed phase column (Acclaim PepMap RSLC, 75 μm x 50 cm (C18, 2 μm, 100 Å) ThermoFisher cat. # 164536) using a gradient of 5% to 35% B in 180 min (Solvent A: 5% acetonitrile/ 0.1% formic acid; Solvent B: 80% acetonitrile/ 0.1% formic acid) at a flow rate of 300 nL/min.

#### Database Searching and Quantitation

Mass spectra were acquired in data-dependent mode with a high resolution (60,000) FTMS survey scan, mass range of m/z 375-1575, followed by tandem mass spectra (MS/MS) of the most intense precursors with a cycle time of 3 s. The automatic gain control target value was 4.0e5 for the survey MS scan. HCD fragmentation was performed with a precursor isolation window of 1.6 m/z, a maximum injection time of 50 ms, and HCD collision energy of 35%. Monoisotopic-precursor selection was set to “peptide.” Precursors within 10 ppm mass tolerance were dynamically excluded from resequencing for 15 s. Advanced peak determination was not enabled. Precursor ions with charge states that were undetermined or >6 were excluded: The raw data are searched with Proteome Discoverer 1.4 against a reviewed human database containing 20,386 protein sequences downloaded October 2021. Variable modification of methionine oxidation was included. Static cysteine modification for carbamidomethyl are also included. Up to 2 missed cleavages are allowed with trypsin selected as the enzyme. Protein identifications were filtered to have an Xcorr vs. charge state >1.5, 2.0, 2.5 for +1, +2, and +3 ions, with at least 2 unique peptides matching the protein, 10 ppm peptide mass accuracy, and 0.6Da fragment ion mass accuracy to be considered a positive identification.

### Statistics

**Figure 1:**
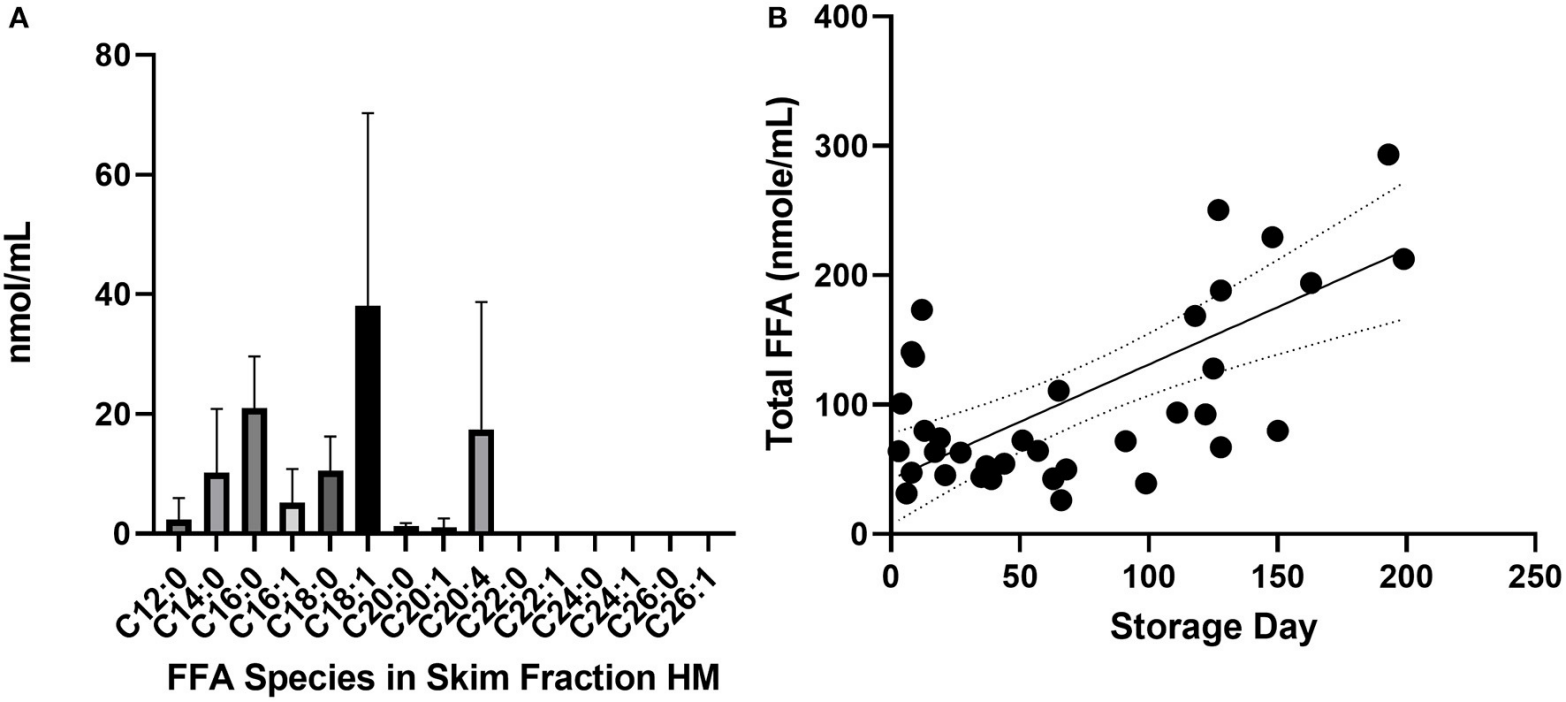
Common species of FFA present in skimmed milk. **(A)** Average concentration of FFA species in undiluted skimmed human milk samples analyzed by UHPLC-tandem MS. Error bars show standard deviation (a) and dotted lines are 95% C.I. around the mean (b). Long chain fatty acids longer than C20 were <1 nmol/mL and below the limit of detection. **(B)** Total FFA content after stored at −20°C, *p* ≤ 0.0001, *n* = 34 samples, 15 mothers. FFAs, free fatty acids; UHPLC, ultra high performance liquid chromatography; MS, mass spectrometry.

This was a pilot study of 34 total milk samples, 55 viability tests (Figure 1) from 15 lactating mothers. This study was designed *a priori* with of *n* = 15 with 3 samples from preliminary data from 3 term mothers. It was powered to observe a 30% loss of viability over 6 months with 99.9% assurance the effect would be seen if it existed. All mothers milk samples obtained were tested for viability. Free fatty acid testing (*n* = 39) was performed after viability testing. Statistics were performed by SPSS v. 27.0 and Prism^®^ 9 GraphPad. Parametric data were analyzed by *t*-tests, and non-parametric tests between related pairs within an experimental condition were analyzed by a Wilcoxon sign rank test. Multiple comparisons with ANOVA were corrected with *post-hoc* Tukey analysis. Linear regression demonstrated by a line on the graphical images was performed by GraphPad with dotted lines indicating 95% confidence intervals. Linear regression for the variables of FFA species on cytotoxicity used the enter-method, and included adjustment for batch variability in SPSS. The dependent variables passed the Kolmogrovo-Smirnov normality test and *F*-test for homoskedasticity. Significance was set at *p* < 0.05.

## Results

Fifteen mothers of infants admitted to the Neonatal Intensive Care Unit at the MUSC Shawn Jenkins Children's Hospital were enrolled to donate their milk for this study. Average maternal age at enrollment was 31 ± 5 years. Eleven mothers were Caucasian and four were Black. One mother had an infant who was term (39 weeks), the other infants were preterm <37 weeks. Their infants had a mean birth gestational age of 28 ± 4 weeks and a birth weight of 1,154 ± 645 g. Fourteen deliveries were singleton, one was a twin delivery. Two or three milk samples were obtained from each mother (Supplementary Figure 1). Milk samples were stored in a freezer at −20°C and were tested over a range of storage days, spanning 5–150 days (Supplementary Figure 2). Median frozen storage time before analysis was 62 days (IQR 38–87). The median post-partum day of expressed milk was 47 days (IQR 15–64.5). After including storage days, there was no correlation between lactation stage and cytotoxicity in a linear regression model.

**Figure 2:**
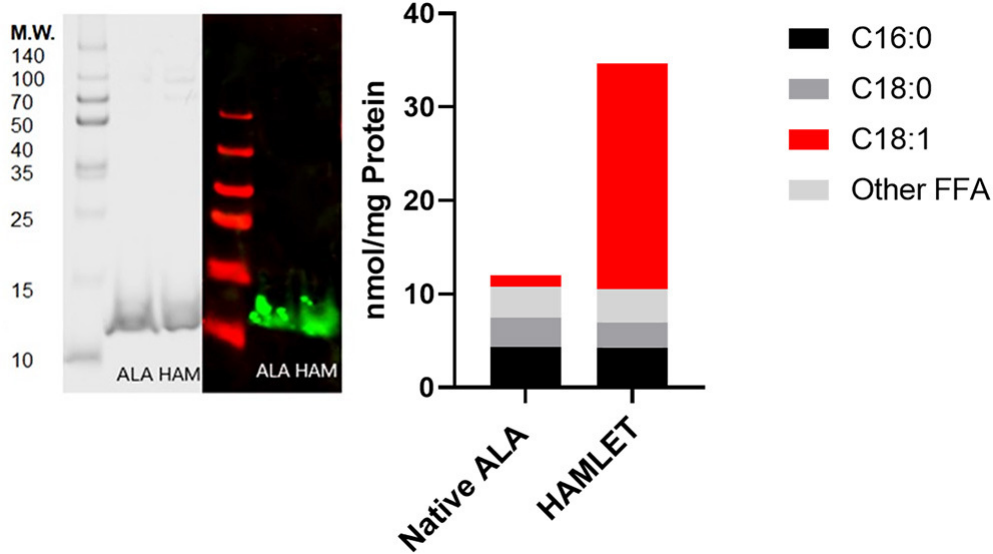
HAMLET verification with SDS-PAGE gel, Western blot and uHPLC. Left: SDS-PAGE gel with Coomassie stain and Western blot for native (purified) α-lactalbumin and HAMLET protein. Right: Free fatty acid content per mg protein analyzed by UHPLC-MS/MS comparing substrate free fatty acid (nmol) associated with mass (mg) of native α-lactalbumin and HAMLET after oleic-acid conditioned anion-exchange chromatography. ALA, α-lactalbumin; HAM, HAMLET.

The most common FFA bound to skimmed milk proteins were C18:1 (oleic acid), C16:0 (palmitic acid), C20:4 (arachidonic acid) and C18:0 (stearic acid) (Figure 1). Fatty acids with chains longer than C22:0 were not highly bound with milk protein in this experiment. Total free fatty acid content of skimmed milk rose significantly over time (Figure 1B, *p* < 0.0001). The protein and oleic acid content of HAMLET and identity of α-lactalbumin in both species were confirmed using Western blot, HPLC (Figure 2) and mass spectrometry (Table 1).

**Table 1:** Proteomic comparison of *HAMLET* and native α-lactalbumin protein.

**HAMLET**	**PSM**	**α-lactalbumin**	**PSM**
NIcDIScDKFLDDDITDDIMcAK	121	NIcDIScDKFLDDDITDDIMcAK	207
ILDIKGIDYWLAHK	234	ILDIKGIDYWLAHK	227
NIcDIScDKFLDDDITDDIMcAKK	128	NIcDIScDKFLDDDITDDIMcAKK	260
ALcTEKLEQWLcEKL	174	ALcTEKLEQWLcEKL	220
KILDIKGIDYWLAHK	102	KILDIKGIDYWLAHK	79
FLDDDITDDIMcAK	6	FLDDDITDDIMcAK	56
ALcTEKLEQWLcEK	14	ALcTEKLEQWLcEK	47
		LEQWLcEKL	34
NIcDIScDKFLDDDITDDImcAK	26	NIcDIScDKFLDDDITDDImcAK	46
cELSQLLK	28	cELSQLLK	39
FLDDDITDDImcAKK	6	FLDDDITDDIMcAKK	38
GIDYWLAHK	23	GIDYWLAHK	31
FLDDDITDDIMcAK	6	FLDDDITDDImcAK	27
cELSQLLK	28	QFTKcELSQLLK	18
NIcDIScDKFLDDDITDDImcAKK	9	NIcDIScDKFLDDDITDDImcAKK	19
FLDDDITDDIMcAKK	15	FLDDDITDDImcAKK	15
ILDIKGIDYWLAHKALcTEK	11	ILDIKGIDYWLAHKALcTEK	7
GIDYWLAHKALcTEK	9	GIDYWLAHKALcTEK	7
GIDYWLAHKALcTEKLEQWLcEK	4	GIDYWLAHKALcTEKLEQWLcEK	6
FLDDDITDDIMcAKKILDIK	1	FLDDDITDDIMcAKKILDIK	5
LWcKSSQVPQSR	2	LWcKSSQVPQSR	4
		SSQVPQSR	1

**Figure 3:**
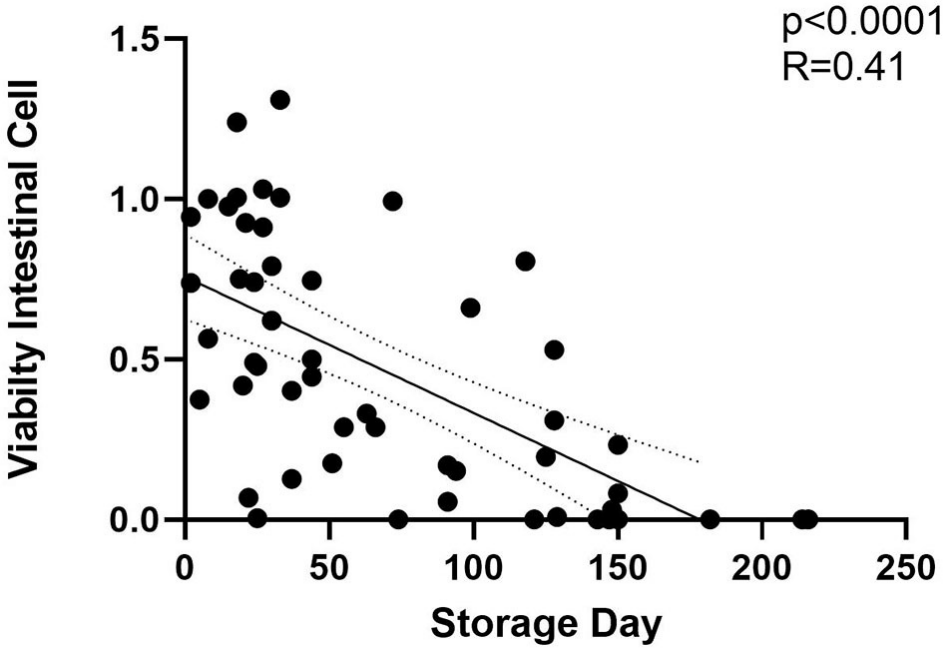
Fetal intestinal cell viability decreases with storage time −20°C. Storage time is in days for both HIEC-6 and FHs 74 Int cell lines, normalized. Includes milk viability tests *n* = 55 from 34 unique milk samples. Significant inverse relationship between cell viability after human milk exposure and time in frozen storage at −20°C. LD50 at 60 days is shown in the horizontal dotted line which is the time point at which 50% of samples killed 50% of cells in culture. Curved dotted line represents 95% C.I. of linear regression slope. Simple linear regression by GraphPad Prism 9 (*p* < 0.001).

**Figure 4:**
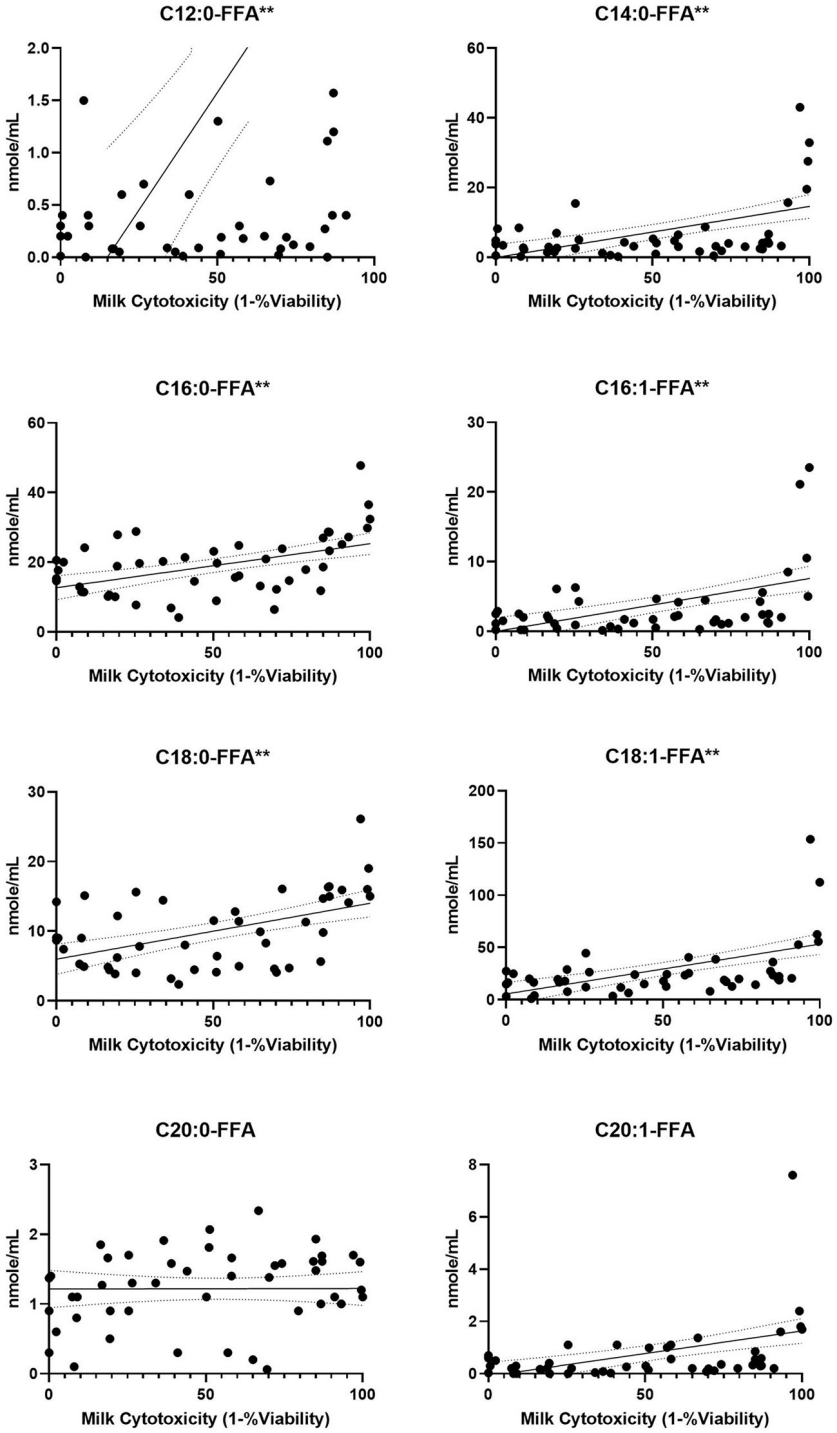
FFA content from human milk fractions increases with cytotoxicity. Previously frozen preterm skimmed (*n* = 34), casein (*n* = 11) and whey (*n* = 11) milk samples were tested at 10% concentrations for intestinal cell viability, then 15 FFA species were quantified by UHPLC-MS/MS. The 8 species shown had concentrations >1 nmol/mL. Simple regression slopes with 95% confidence intervals are shown. Significance noted as **p < 0.0001. FFA, free fatty acids; UHPLC, ultra high performance liquid chromatography; MS, mass spectrometry.

**Figure 5:**
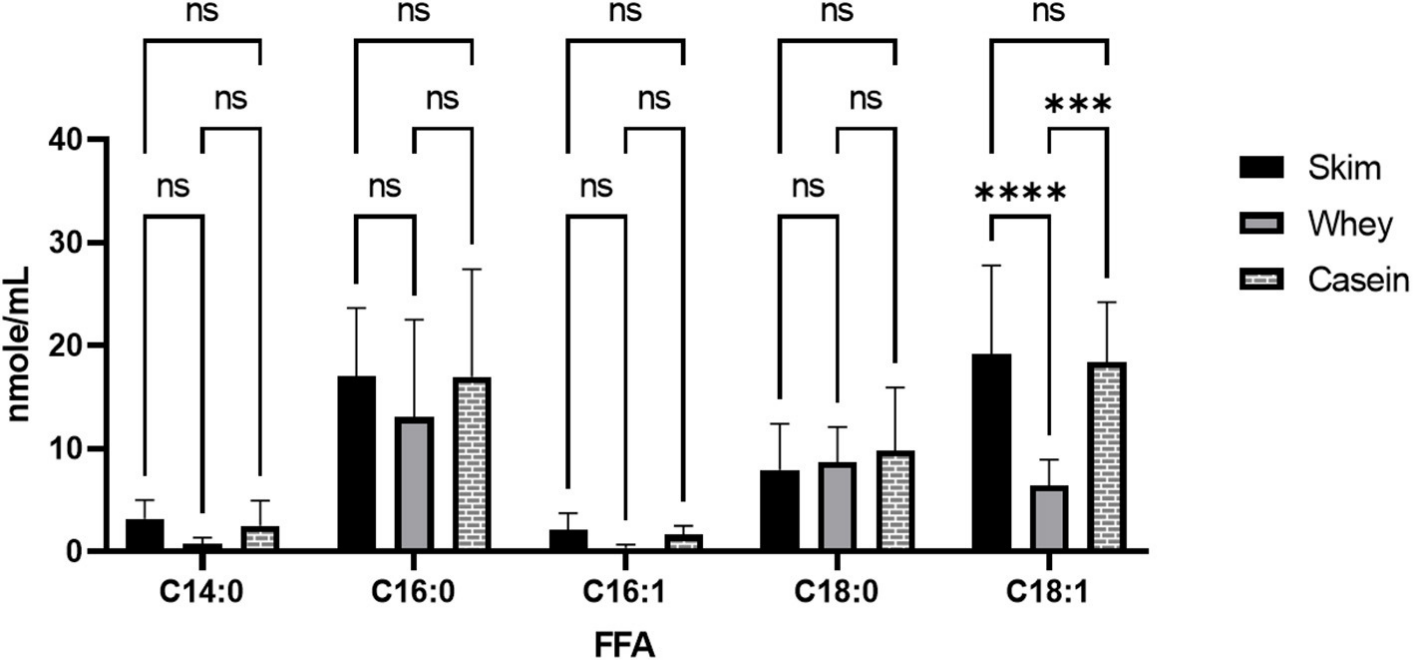
Free oleic acid in casein vs. whey fractions in human milk. Milk (*n* = 6) was skimmed, 1.0N HCl was added to adjust milk pH to 4.3. Acidified skimmed milk was centrifuged at 8000 × g for 10 min to precipitate casein from whey layer. Separated proteins were pH corrected to 6.8 ± 0.1 pH in HBSS and analyzed for FFA content using UHPLC-MS/MS. GraphPad Prism calculated ANOVA tests with *post-hoc* Tukey multiple comparisons tests, significance *p* < 0.005***, *p* < 0.0001**** or NS (non-significant). HBSS, Hank's buffered salt solution; FFA, free fatty acid; UHPLC-MS/MS, Ultra high performance liquid chromatography coupled with tandem mass spectrometry.

Each milk sample was skimmed and analyzed for cytotoxicity in fetal intestinal cell cultures. The observations of cell viability loss after exposure to milk increased in frequency and severity with respect to time in frozen storage (Figure 3). Using linear regression, with a 95% C.I. to interpolate unknowns, the LD50 occurred at an average 54 days (34–72 days) in frozen storage, in which toxicity to at least 50% or more of cells in culture was seen more than 50% of the time. At the time of viability testing, defatted milk was analyzed for FFA content. There was a direct relationship with the major species of protein-associated FFA and milk cytotoxicity against intestinal cells (Figure 4). Oleic acid (C18:1) was significantly associated with cytotoxicity (*p* < 0.0001). Within an enter-method (variables equally weighted) multivariable linear regression model, including all FFA species measured, only oleic fatty acid remained significantly associated with cytotoxicity (*p* = 0.038, SPSS v.27, Supplementary Figure 3). When the most prevalent 5 fatty acids were included alone, the association between OA and cytotoxicity was more significant (*p* = 0.009). This model corrected for differences between viability testing batches (Supplementary Figure 4). After acid-precipitation of 6 cytotoxic samples, oleic acid was higher in the casein fraction than the whey fraction (Figure 5), and follow-up viability testing showed more toxicity in the casein (57%) vs. whey fractions (24%) *p* < 0.0001, (% cell death by MTT). Only C18:1 fatty acid species significantly localized to the casein fraction, but not other prevalent saturated or unsaturated fatty acids (Figure 5).

**Figure 6:**
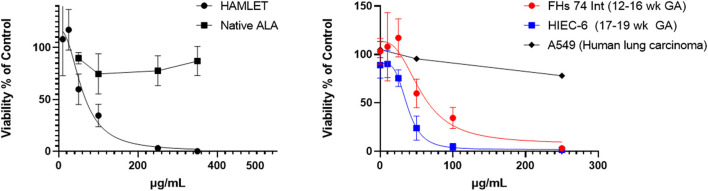
HAMLET vs. α-lactalbumin cytotoxicity in fetal intestinal cells. Left: Comparison of HAMLET with native substrate alpha-lactalbumin. Right: Comparison of 2 non-transformed fetal epithelial intestinal cell lines (Fhs 74, HIEC-6) and a lung carcinoma cell line (A549) with increasing concentrations of HAMLET. The estimated lethal dose of HAMLET to kill 50% of cells in culture (LD50) is 56.9 (34.1–79.4) μg/mL for FHs 74 Int and HIEC-6 has an LD50 of 37.6 (30.97–44.2) μg/mL. Viability was measured with MTT assay after 3.5 h. Dotted lines are average curves plotted by GraphPad Prism 9. All data are presented as mean ± SD (95 C.I.).

**Figure 7:**
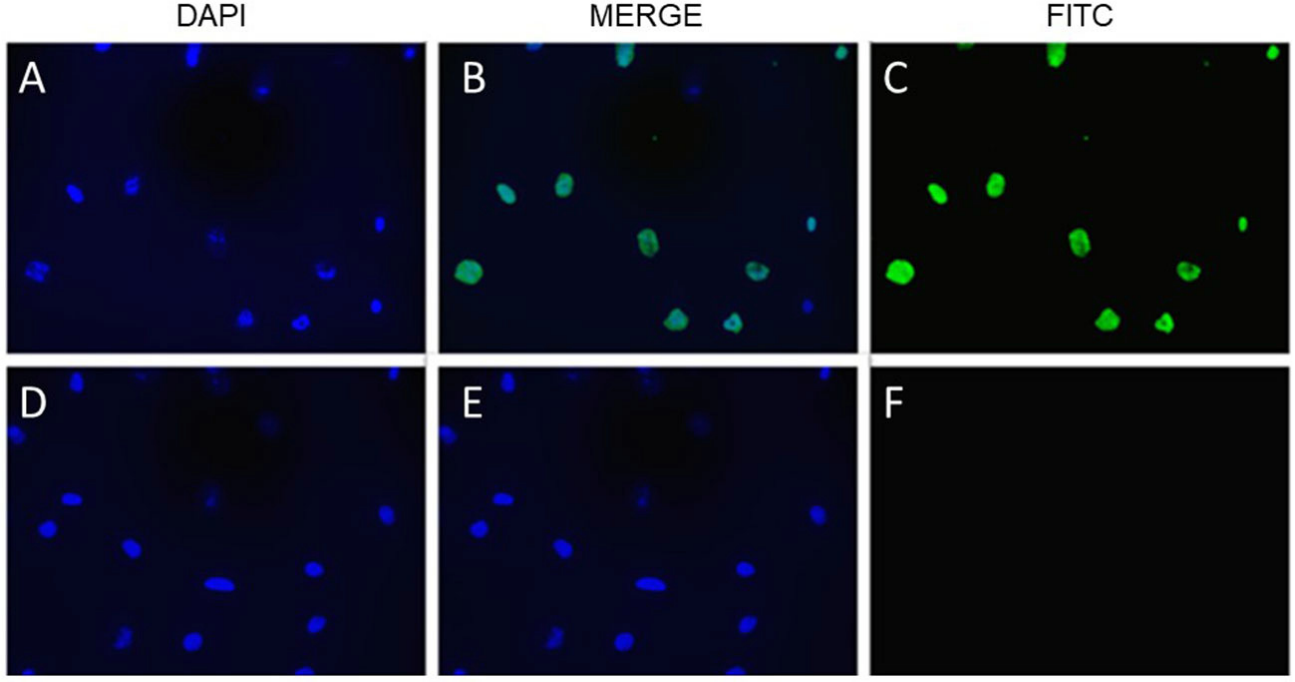
Cytotoxic milk and apoptosis in fetal intestinal cells. TUNEL staining of FHs 74 cells, with DAPI stain of cells exposed to milk 92 days old **(A)**, FITC stain **(C)** and merged image **(B)**. **(D–F)** are DAPI, merged and FITC stain of cells exposed to milk 40 days old. Bottom row are cells exposed to milk 40 days old. Fluorescent microscopy of cells mounted on glass slides.

As shown in Figure 6, the laboratory-synthesized α-lactalbumin-oleic acid complex (HAMLET) was cytotoxic to both cells in monoculture. Alpha-lactalbumin and HAMLET were also verified by mass spectrometry to have identical sequences (Table 1), and HPLC MS/MS verified the presence of increased OA on the HAMLET complex. Synthesized HAMLET was cytotoxic to HIEC-6 with LD50 of 37.6 (30.97–44.2 μg/mL) and FHs 74 with LD50 of 56.9 (34.1–79.4 μg/mL). HIEC-6 was more sensitive to HAMLET cytotoxicity than FHs 74 in identical experimental conditions (*p* = 0.01 Wilcoxon Sign Test, Figure 6B). Native α-lactalbumin was not cytotoxic to intestinal cells, and the A549 cell line was the least sensitive to HAMLET. To investigate the type of cell-death seen in milk cytotoxicity, a 96-day-old sample of human milk (confirmed first by MTT) was diluted to 1% concentrations and incubated for 2 h with FHs 74 grown on slides and TUNEL stained (Figure 7). Apoptosis, rather than lysis, was demonstrated. Non-cytotoxic human milk (confirmed by MTT) was used as a comparison, and it did not promote apoptosis.

## Discussion

Fatty acid cytotoxicity and free oleic-acid induced injury may be detrimental to the intestine of preterm infants, who have under-developed epithelial barriers and low mucin levels (36). Free oleic acid can uncouple oxidative phosphorylation (37), slowing the growth of rapidly proliferating cells. Excess FFA in the diet of preterm infants could directly affect the growing fetal intestine. In the fetal pig, intestinal growth exceeds both the rate of total body weight gain, and increases in masses for the liver, heart, kidney and brain in the same period (38)–making it the most rapidly growing internal organ in the fetus and thus highly susceptible to cytotoxic agents. It is known that free oleic acid can be detrimental to intestinal epithelium, but previous animal studies used much higher doses of FFA. A study by Ishikawa et al. showed that EGF attenuated the 40 mM doses of oleic-acid induced injury on rat jejunal cells through the mechanism of EGF-stimulated mucus production (39). This study is the first to show that even micromolar concentrations (15 μM) of oleic acid coupled to the milk protein layer can kill fetal epithelial cells in culture.

Furthermore, the oleic-acid linked cytotoxicity was apparent in the vast majority of milk samples after 2 months of storage, a significantly shorter window of time than previously known. For reference, current CDC guidelines state that human milk can be stored safely frozen at −20°C for up to 12 months. While this empiric practice has no known adverse effects for the term infant, questions of safety arise for the very preterm population. Importantly, we did not observe any cytotoxicity as a feature in fresh-frozen milk, which indicates that a biochemical process is occurring over time. It is well-known that lipases remain active, even in −20°C storage conditions (24). We speculate that active milk lipases may be contributing to the rise of FFA over time and lead to the formation of bioactive protein-lipid complexes that can be detected in the skimmed layer of milk. In a linear regression model that included 15 FFA using cytotoxicity as a dependent variable, we found that oleic acid but not other species is significantly associated with this cytotoxicity. Interestingly, the linear model of only the most prevalent FFA species, we found that arachidonic acid was significantly associated with increased viability (Supplementary Figure 4). While FFA from milk has been known to induce toxicity in various cell types (20), this study reports the novel finding that milk oleic acid is playing a key role in FFA cytotoxicity for non-cancerous immature intestinal cells.

Similar to previous studies (27), cytotoxicity was observed in both the skimmed and whole milk, indicating that FFA remained present in defatted layers, bound to protein. We measured protein-lipid complexes by using complete organic extraction of FFA from the skimmed milk protein layer before analysis. Furthermore, using skimmed milk for viability testing yielded more precise and repeatable MTT assay results. Specifically, we avoided spectrophotometric interference related to opaque milk fat. This finding supports the role of fatty acid-protein complexes in cytotoxic bioactivity.

The most-studied cytotoxic milk-derived complex, HAMLET, has not been shown to be cytotoxic to some mature non-transformed cells, but has cytotoxicity in many cancerous cell lines, lymphocytes, immature cells, and stem cells (26, 27, 40). Therefore, we speculate that HAMLET may be forming in milk over time and playing a role in the observed milk cytotoxicity. To confirm that HAMLET could cause fetal intestinal cell cytotoxicity, we synthesized and confirmed HAMLET's ability to cause cytotoxicity in the same cell lines. However, more work is needed to determine if HAMLET alone is contributing to cytotoxicity, rather than other cytotoxic factors (specifically other oleic-acid bound proteins). Oleic acid is highly associated with bulk casein layer proteins, which suggests that oleic acid may be binding to casein proteins as well as to α-lactalbumin. The degree of cytotoxicity related to α-lactalbumin bound oleic acid, rather than other proteins (e.g., casein) needs further study.

We suggest that the cytotoxicity found in skimmed milk is due to HAMLET-like proteins, in which multiple types of protein may be behaving similarly to HAMLET. When the oleic acid concentration per mg protein (OA nmol per protein mg) within both casein and whey were compared—they showed similar toxicity, supporting the idea that cytotoxicity is a feature of the OA content, rather than the type of protein. We observed that the synthesized HAMLET complex bound significantly more free oleic acid per mg protein than purified α-lactalbumin (24 vs. 1.2 nmol/mg, respectively). Figure 6 confirmed that HAMLET, but not native (minimal oleic acid bound) α-lactalbumin, was cytotoxic to fetal intestinal cells. It is possible that the α-helical component of protein that couples with oleic acid, is key to generating cytotoxicity (41). If this is the case, lactoferrin, which has significant α-helical content comprised of 27 α-helices, may also have a role. These cytotoxic effects have been previously seen with bovine lactoferrin (rather than α-lactalbumin) coupled with oleic acid, which suggests alternate species within the category of HAMLET-like complexes (42, 43). We do not know how HAMLET is forming spontaneously. Previous studies report that calcium must be fully removed from solution and the α-lactalbumin unfolded before it will bind to oleic acid and form HAMLET (44). It is unclear how HAMLET may form in an equilibrium with high oleic acid in the presence of calcium and available binding proteins. HAMLET is a kinetically stable state, a feature which could drive its spontaneous formation with time (45).

Furthermore, FHs 74 cells were more resistant to HAMLET than HIEC-6. Even though HIEC-6 is a slightly more mature cell line, it was more susceptible to HAMLET. Since FHs 74 cells grow more rapidly than HIEC-6 cells, they were more confluent than HIEC-6 cultures before experimentation when seeded at equivalent numbers. We speculate the result of higher numbers of epithelial cells may be forming a stronger barrier against HAMLET cytotoxicity and cell entry. Additionally, the lethal doses of BAMLET (HAMLET from bovine α-lactalbumin) in monoculture have been previously shown to be highly dependent on cell number (35). We further confirmed that both cytotoxic milk and HAMLET cause apoptosis rather than lysis, similar to the ability of HAMLET's ability to induce apoptosis in other cell models (28). In animal models, oral HAMLET administration reduces intestinal tumor burden in mice (46), which suggests HAMLET can target intestinal cells after ingestion. Due to rapid cell fragmentation at 10%, the milk concentration was lowered 1% to capture the occurrence of apoptosis before cells detached from the glass slides. Whether milk's cytotoxicity is due to HAMLET or HAMLET-like complexes (i.e., in which various oleic acid binding proteins like casein or lactoferrin) (43) contribute equally, this cytotoxic phenomenon is also unknown. This study highlights the necessity of studying dietary components in the immature intestine to explore the unique susceptibilities of these tissues. Rigorous research involving tissue models of early developmental stages could improve the understanding of preterm intestinal diseases like necrotizing enterocolitis, or poor somatic growth.

Bovine protein in formula, specifically casein, has been targeted in the pathogenesis of necrotizing enterocolitis (NEC) (47). Even after modest decreases of NEC rates reported in many institutions from human milk diets, necrotizing enterocolitis and other gastrointestinal complications still occur in many extremely preterm infants. Human casein proteins are naturally low in both colostrum and maternal milk expressed early in lactation. We observed that the oleic acid-bound proteins localized to the casein layer after precipitation. It is possible that the clinical association between bovine casein and NEC pathogenesis (48) could be from bound oleic acid within the casein. Avoiding bovine products, specifically formula, has become central dogma for many efforts aimed toward (49) reducing necrotizing enterocolitis (50, 51). Interestingly, modern infant formula contains chemically modified high-oleic acid safflower oil, which has been shown to impart a higher load of oleic acid in the intestine as previously shown with *in vitro* digestion using pancreatic lipases (21). Necrotizing enterocolitis remains a multifactorial disease that may be driven by both prematurity, microbial influences and inflammation from allergic responses bovine proteins and/or FFA-related cytotoxicity.

Limitations of this study include not using digestive enzymes to pre-digest milk in this study. The early preterm intestinal environment is dependent on diet and gestational age (27). In general, preterm infants produce lower quantities of bile salts, pancreatic enzymes, proteases, and gastric lipases than term infants (52). Activities of many gastric and pancreatic enzymes also are reduced in preterm neonates (23). Preterm infants depend largely on the action of human milk bile-stimulated lipases to compensate for low endogenous enzymes. Additionally, the gastric pH of a preterm infant during feeding remains relatively neutral up to the first hour (49), only becoming acidic between feedings. Maturation of digestion and increased acid secretion occurs gradually over the first months of life (53). The activity of pH-dependent pepsins on the milk itself is difficult to extrapolate. For these reasons, this study did not attempt to recapitulate the gastric conditions of early preterm infants with an *in vitro* milk digestion step. However, a model incorporating these enzymes would further the understanding of how HAMLET may affect epithelium *in vivo*.

Importantly, there are limitations to studying cytotoxicity within cellular cultures. HAMLET may not impart the same cytotoxicity in more complex tissue models or in the mature neonatal intestine. In fact, no major negative effects have been documented in term healthy infants ingesting their own mother's milk after prolonged freezing up to a year. Also, there were caveats to this FFA analysis. This analysis did not quantify free eicosapentaenoic acid, 20:5(n-3), and docosahexaenoic acid (DHA) 22:6(n-3) which is also present in human milk. Additionally, the *cis* vs. *trans* configurations and the respective positions of the unsaturated fatty acid bonds were not determined and may modify cytotoxicity. For example, the most prevalent species C18:1:9*cis* confirmation has shown cytotoxicity in previous studies, but not the rare forms of C18:1:9*trans* or C18:1:11*cis*. It should also be noted that the time period suggested as the lethal dosage of 50% is based on study *in vitro* culture conditions and are not yet ready to be extrapolated to clinical practice (e.g., how long or in what other conditions to store mother's milk) unless used as time-points are confirmed in further clinical studies. The type of milk tested in this study was skimmed milk because the cellular washes needed with whole milk additions increased variability in the viability assays. Yet, skimmed milk samples and whole milk samples showed excellent agreement with cytotoxicity based on age when tested as either their whole component or skimmed at 10% concentrations (*n* = 6, Pearson *r* = 0.9859, C.I. 0.8722–0.9985). Further clinical research is needed to determine the biological implications of storage age on the very preterm infant intestinal environment. It is further unknown if HAMLET-like complexes may form in refrigerated storage conditions after freezing, as previous studies show FFA also rise in milk at 4°C (11).

In summary, after frozen storage, milk was cytotoxic to intestinal cells. This effect was associated with protein-bound oleic acid, and the effect increased with time. Most milk samples showed some degree of cytotoxicity if stored frozen for sufficient lengths of time. Synthesized HAMLET was confirmed to induce immature intestinal cell cytotoxicity in monoculture. Non-specific binding of free oleic acid to various aqueous proteins or α-lactalbumin could explain the observed cytotoxicity of stored milk on intestinal cells by the generation of HAMLET-like complexes. Until appropriate studies are performed using human tissue explants, animal models and targeted, or prospective observational human studies, we caution against extrapolating clinical implications from this study. There is a major gap in understanding how the immature rapidly growing intestine responds to HAMLET and other complex nutrients. Why growing or undifferentiated intestinal epithelial tissue may be susceptible to nutrient-related cytotoxicity is a needed area of study that may inform the best practices for feeding very preterm infants.

## Data Availability Statement

The datasets presented in this study can be found in online repositories. The names of the repository/repositories and accession number(s) can be found in the article/Supplementary Material.

## Ethics Statement

The studies involving human participants were reviewed and approved by Medical University of South Carolina IRB. The patients/participants provided their written informed consent to participate in this study.

## Author Contributions

KC, JB, and CW: concept and design. JB, CW, KC, and DN: intellectual contribution. KC: sample collection. JB, KC, and DN: data acquisition. JB, KC, and CW: data analysis, statistical analysis, interpretation, drafting, and editing manuscript. KC and CW: obtained funding. All authors contributed to the article and approved the submitted version.

## Funding

This publication was supported, in part, by the National Center for Advancing Translational Sciences of the National Institutes of Health under Award Numbers KL2TR001452 (KC) & UL1TR001450 (KC and CW). The content is solely the responsibility of the authors and does not necessarily represent the official views of the National Institutes of Health. This study is also supported by the David and Laura Stone Endowment for Advancement in Neonatal Medicine, Charleston, South Carolina (KC and CW). This study was also supported by the Lipidomics Shared Resource, Hollings Cancer Center, Medical University of South Carolina (P30 CA138313 and P30 GM103339). The Proteomics Center is supported by the MUSC Vice President for Research, College of Medicine, Hollings Cancer Center (P30 CA138313), SC COBRE in Oxidants, Redox Balance and Stress Signaling (P20 GM103542), MUSC Digestive Disease Research Core Center (P30 DK123704), NCI/Alliance of Glycobiologists for Cancer Research, the NIH Shared Instrumentation Grant Program, and the South Carolina Centers of Economic Excellence Program.

## Conflict of Interest

The authors declare that the research was conducted in the absence of any commercial or financial relationships that could be construed as a potential conflict of interest.

## Publisher's Note

All claims expressed in this article are solely those of the authors and do not necessarily represent those of their affiliated organizations, or those of the publisher, the editors and the reviewers. Any product that may be evaluated in this article, or claim that may be made by its manufacturer, is not guaranteed or endorsed by the publisher.

## Supplementary Material

Supplementary Figure 1 Flow chart of patient enrollment, sample collection and testing. Mothers (*n* = 15) donated milk samples (*n* = 34), which were repeatedly tested (*n* = 55) until cytotoxicity was observed. All tests observed with corresponding storage days. One sample had 4 viability tests.

Supplementary Figure 2 Range of milk samples. Preterm mothers (*n* = 15) donated milk samples (*n* = 34) over batches donated (1st, 2nd, and 3rd). Samples covered a range of storage times before viability testing, ranging from 2 days to 199 days.

Supplementary Figure 3 Cytotoxicity and FFA species present. In a simple linear regression model of FFA concentration vs. cytotoxicity without adjustment. All subjects (*n* = 15), viability tests *n* = 55 were included in this model. Total FFA were not included in the model due to rejection secondary co-linearity with C18:1 (variance 0.82). SPSS 27.0, *p* < 0.05 is significant.

Supplementary Figure 4 Summary of linear regression modeling. Coefficients per FFA species and cytotoxicity adjusted for batch effects and selected for the 5 most prevalent FFA concentrations. All subjects (*n* = 15) with HPLC-MS/MS FFA analysis coupled with viability tests (*n* = 39) were included in this model. Enter-method regression with SPSS 27.0, *p* < 0.05 is significant.
