# Supplementary material for: Free Fatty Acid and α-Lactalbumin-Oleic Acid Complexes in Preterm Human Milk Are Cytotoxic to Fetal Intestinal Cells in vitro
Source: Front Nutr. 2022 Jul 5;9:918872. doi: 10.3389/fnut.2022.918872 (PMC9294382; doi:10.3389/fnut.2022.918872)
Supplement: Supplementary Figure 1 — Flow chart of patient enrollment, sample collection and testing. Mothers (n = 15) donated milk samples (n = 34), which were repeatedly tested (n = 55) until cytotoxicity was observed. All tests observed with corresponding storage days. One sample had 4 viability tests. [file Presentation_1.pdf]

Supplemental Figure 1

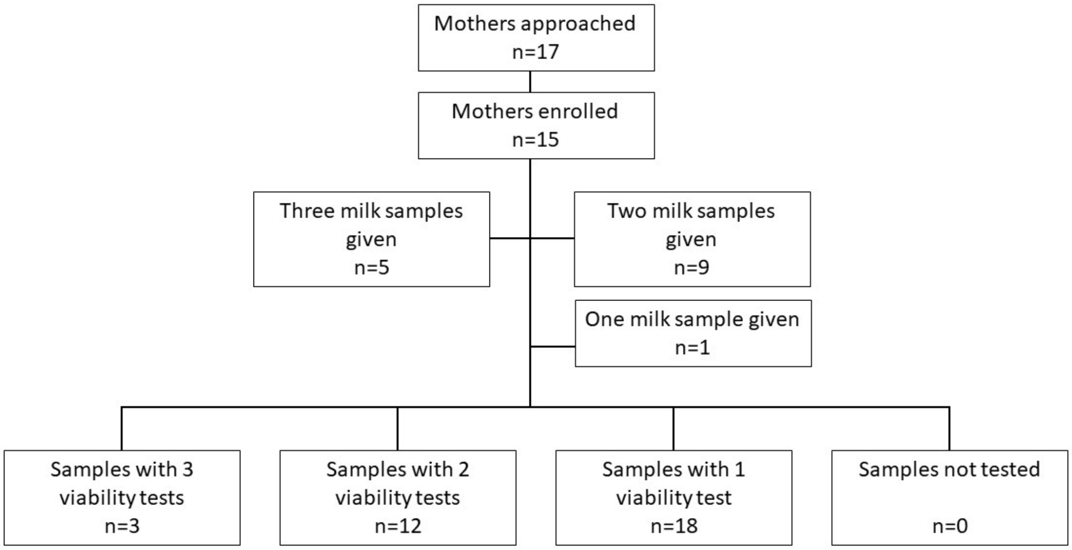

Figure S1. *Flow chart of patient enrollment, sample collection and testing*. Mothers (n=15) donated milk samples (n=34) which were repeatedly tested (n=55) until cytotoxicity was observed. All tests observed with corresponding storage days. One sample had 4 viability tests.

Supplemental Figure 2.

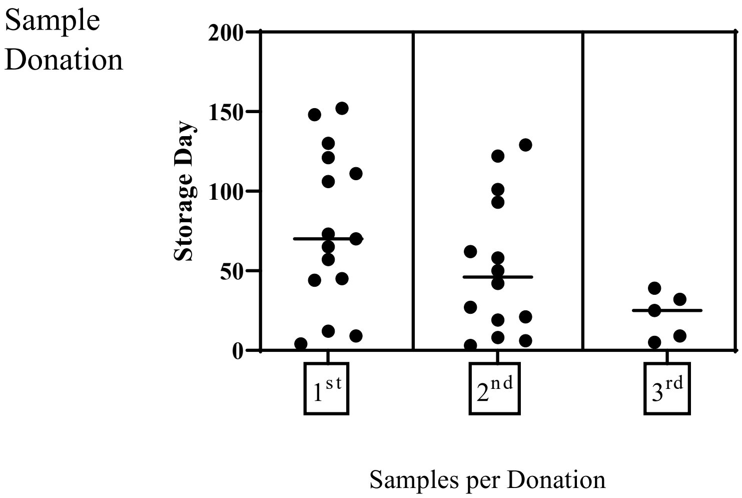

Figure S2. *Range of milk samples.* Preterm mothers (n=15) donated milk samples (n=34) over batches donated (1st, 2nd and 3rd). Samples covered a range of storage times before viability testing, ranging from 2 days to 199 days.

Supplemental Figure 3.

Coefficients[a]

Model		Unstandardized Coefficients B	Unstandardized Coefficients Std. Error	Standardized Coefficients Beta	t	Sig.	Collinearity Statistics Tolerance	Collinearity Statistics VIF
1	(Constant)	8.369	23.538		.356	.725		
	C120FFA	-3.834	4.611	-.361	-.831	.414	.067	14.837
	C140FFA	1.476	2.240	.412	.659	.516	.032	30.827
	C160FFA	.549	1.376	.125	.399	.694	.129	7.737
	C161FFA	-.317	4.331	-.047	-.073	.942	.031	31.939
	C180FFA	-.717	2.318	-.108	-.309	.760	.104	9.607
	C181FFA	1.802	.817	1.523	2.207	.038	.027	37.584
	C200FFA	-13.497	12.443	-.173	-1.085	.289	.498	2.009
	C201FFA	-3.149	16.943	-.121	-.186	.854	.030	33.350
	C204FFA	-1.136	1.113	-.634	-1.020	.318	.033	30.401
	C220FFA	-65.172	662.693	-.032	-.098	.923	.117	8.525
	C221FFA	288.158	321.827	.559	.895	.380	.033	30.730
	C240FFA	889.100	879.295	.643	1.011	.322	.031	31.936
	C241FFA	-561.776	378.000	-.964	-1.486	.151	.030	33.174
	C260FFA	-1058.199	1992.205	-.329	-.531	.600	.033	30.225
	C261FFA	410.882	825.269	.193	.498	.623	.085	11.814

a. Dependent Variable: Cytotoxicity

Figure S3. *Cytotoxicity and FFA species present.* In a simple linear regression model of FFA concentration vs. cytotoxicity without adjustment. All subjects (n=15), FFA-coupled viability testing (n=39) were included in this model. Total FFA were not included in the model due to rejection secondary to co-linearity with C18:1 (variance 0.82). Enter-method regression SPSS 27.0, $p < 0.05$ is significant.

Supplemental Figure 4.

Model Summary

Model	R	R Square	Adjusted R Square	Std. Error of the Estimate	Change Statistics				
					R Square Change	F Change	df1	df2	Sig. F Change
1	.730[a]	.533	.462	27.95713	.533	7.523	5	33	<.001
2	.772[b]	.596	.520	26.40394	.063	4.997	1	32	.033

a. Predictors: (Constant), C204FFA, C180FFA, C160FFA, C140FFA, C181FFA

b. Predictors: (Constant), C204FFA, C180FFA, C160FFA, C140FFA, C181FFA, Batch

Coefficients[a]

Model		Unstandardized Coefficients		Standardized Coefficients	t	Sig.	Collinearity Statistics	
		B	Std. Error	Beta			Tolerance	VIF
1	(Constant)	15.184	14.054		1.080	.288		
	C140FFA	1.657	1.324	.462	1.252	.220	.104	9.641
	C160FFA	-.160	1.208	-.036	-.132	.896	.187	5.338
	C180FFA	1.200	1.744	.181	.688	.496	.206	4.865
	C181FFA	1.068	.484	.903	2.207	.034	.085	11.816
	C204FFA	-1.501	.685	-.837	-2.190	.036	.097	10.322
2	(Constant)	62.403	24.948		2.501	.018		
	C140FFA	2.565	1.315	.716	1.951	.060	.094	10.660
	C160FFA	-.801	1.177	-.182	-.681	.501	.176	5.675
	C180FFA	.463	1.679	.070	.276	.784	.198	5.060
	C181FFA	1.316	.470	1.112	2.798	.009	.080	12.511
	C204FFA	-1.638	.650	-.914	-2.520	.017	.096	10.415
	Batch	-17.578	7.864	-.346	-2.235	.033	.526	1.901

a. Dependent Variable: Cytotoxicity

Figure S4. Summary of linear regression modeling. Coefficients per FFA species and cytotoxicity adjusted for batch effects and selected for the 5 most prevalent FFA concentrations. All subjects (n=15) with HPLC-MS/MS FFA analysis coupled with viability tests (n=39) were included in this model. Enter-method regression with SPSS 27.0, $p < 0.05$ is significant.
